# Performance of gene-expression profiling test score variability to predict future clinical events in heart transplant recipients

**DOI:** 10.1186/s12872-015-0106-1

**Published:** 2015-10-09

**Authors:** Maria G. Crespo-Leiro, Jörg Stypmann, Uwe Schulz, Andreas Zuckermann, Paul Mohacsi, Christoph Bara, Heather Ross, Jayan Parameshwar, Michal Zakliczyński, Roberto Fiocchi, Daniel Hoefer, Mario Deng, Pascal Leprince, David Hiller, Lane Eubank, Emir Deljkich, James P. Yee, Johan Vanhaecke

**Affiliations:** Instituto de Investigación Biomédica de A Coruña (INIBIC), Complexo Hospitalario Universitario de A Coruña (CHUAC), SERGAS. Universidade da Coruña (UDC), Coruña, Spain; University Hospital Muenster, Muenster, Germany; Ruhr University of Bochum, Bad Oeynhausen, Germany; Medical University of Vienna, Vienna, Austria; University Hospital Bern, Bern, Switzerland; Hannover Medical School, Hannover, Germany; Toronto General Hospital, Toronto, Canada; Papworth Hospital, Papworth Everard, Cambridge, UK; Silesian Center for Heart Disease, Zabrze, Poland; Ospedali Riuniti di Bergamo, Bergamo, Italy; Innsbruck Medical University, Innsbruck, Austria; David Geffen School of Medicine, University of California, Los Angeles, USA; Groupe Hospitalier Pitié-Salpêtrière, Paris, France; CareDx, Brisbane, USA; University Hospital of Leuven, Leuven, Belgium; Department of Cardiology, Herestraat 49, 3000 Leuven, Belgium

**Keywords:** Heart transplant, Gene expression profiling, AlloMap, Surveillance of cardiac recipients, Acute cellular rejection, AlloMap score variability, Gene expression profiling score

## Abstract

**Background:**

A single non-invasive gene expression profiling (GEP) test (AlloMap®) is often used to discriminate if a heart transplant recipient is at a low risk of acute cellular rejection at time of testing. In a randomized trial, use of the test (a GEP score from 0–40) has been shown to be non-inferior to a routine endomyocardial biopsy for surveillance after heart transplantation in selected low-risk patients with respect to clinical outcomes. Recently, it was suggested that the within-patient variability of consecutive GEP scores may be used to independently predict future clinical events; however, future studies were recommended. Here we performed an analysis of an independent patient population to determine the prognostic utility of within-patient variability of GEP scores in predicting future clinical events.

**Methods:**

We defined the GEP score variability as the standard deviation of four GEP scores collected ≥315 days post-transplantation. Of the 737 patients from the Cardiac Allograft Rejection Gene Expression Observational (CARGO) II trial, 36 were assigned to the composite event group (death, re-transplantation or graft failure ≥315 days post-transplantation and within 3 years of the final GEP test) and 55 were assigned to the control group (non-event patients). In this case-controlled study, the performance of GEP score variability to predict future events was evaluated by the area under the receiver operator characteristics curve (AUC ROC). The negative predictive values (NPV) and positive predictive values (PPV) including 95 % confidence intervals (CI) of GEP score variability were calculated.

**Results:**

The estimated prevalence of events was 17 %. Events occurred at a median of 391 (inter-quartile range 376) days after the final GEP test. The GEP variability AUC ROC for the prediction of a composite event was 0.72 (95 % CI 0.6-0.8). The NPV for GEP score variability of 0.6 was 97 % (95 % CI 91.4-100.0); the PPV for GEP score variability of 1.5 was 35.4 % (95 % CI 13.5-75.8).

**Conclusion:**

In heart transplant recipients, a GEP score variability may be used to predict the probability that a composite event will occur within 3 years after the last GEP score.

**Trial registration:**

Clinicaltrials.gov identifier NCT00761787

**Electronic supplementary material:**

The online version of this article (doi:10.1186/s12872-015-0106-1) contains supplementary material, which is available to authorized users.

## Background

In a landmark article published more than 40 years ago, Dr. Philip Caves and colleagues described the percutaneous transvenous endomyocardial biopsy (EMB) and reported their experience with the 67 cardiac biopsies performed in 17 cardiac transplant recipients [1]. Since then, serial EMB has become the standard of care for monitoring transplant rejection status in adult patients [2]. Despite its wide acceptance, EMB has several limitations. First, patients who undergo EMB, particularly those undergoing biopsy surveillance for a prolonged period of time, are at increased risk of complications [3]. Second, the pathological interpretation can be subjective [4, 5]. Third, the procedure can only detect rejection after cellular infiltration and/or graft damage has occurred [5], limiting a prognostic utility that may guide optimization of immunosuppression therapy. Finally, there is a noteworthy cost and patient inconvenience associated with EMB surveillance. Consequently, a significant interest in developing novel surveillance strategies has fueled many attempts at finding alternative non-invasive tests to monitor allograft status [6].

A number of alternative tests have been developed but have either failed to deliver adequate performance or their implementation proved complex [5]. In contrast, in a large randomized controlled trial, a non-invasive gene expression profiling (GEP) test (AlloMap®, CareDx, Brisbane, California) was found to be non-inferior to routine EMB for surveillance after heart transplantation in selected low-risk patients with respect to clinical outcomes [7]. The test is performed on a peripheral blood sample; the results are reported as a single GEP score (range from 0 to 40) [8]. Since the GEP test became available in 2005, more than 69,500 tests have been performed on more than 15,000 unique heart transplant recipients. This surveillance methodology has been generally used in adult patients older than 15 years for identification of heart transplant recipients at a low risk of acute cellular rejection at time of testing [2].

In addition to the diagnostic utility of the single GEP score, Deng and colleagues noted an association between low variability of serial GEP scores and the clinical stability in patients [9]. These initial findings have been independently confirmed by post-hoc multivariate analyses of 602 heart transplant patients enrolled in the Invasive Monitoring Attenuation by Gene Expression Profiling (IMAGE) study [10]. This analysis suggested that the variability of GEP scores from an individual may predict the risk of future allograft dysfunction or death in that individual. The prognostic utility of serial GEP scores in predicting a clinical event was found to be independent of, and complementary to, the original use of a single GEP score in estimating the probability of acute cellular rejection at the time of testing. However, further validation of a serial characteristic of GEP scores was suggested [10].

Therefore, in the current study, we expanded on the earlier findings by conducting an analysis of an independent patient population from the Cardiac Allograft Rejection Gene Expression Observational (CARGO) II study to determine: (1) the prognostic utility of within-patient variability of GEP scores in predicting future significant clinical events; (2) the negative predictive value (NPV) and the positive predictive value (PPV) of GEP score variability in predicting future significant clinical events.

## Methods

### Ethics, consent and permissions

The CARGO II study was approved by local institutional review boards or ethics committees of 12 participating institutions (Instituto de Investigación Biomédica de A Coruña (INIBIC), Complexo Hospitalario Universitario de A Coruña (CHUAC), SERGAS. Universidade da Coruña, Spain; University Hospital Muenster, Muenster, Germany; Medical University of Vienna, Vienna, Austria; Hannover Medical School, Hannover, Germany; Toronto General Hospital, Toronto, Canada; Papworth Hospital, Papworth Everard, Cambridge, United Kingdom; Silesian Center for Heart Disease, Zabrze, Poland; Ospedali Riuniti di Bergamo, Bergamo, Italy; Innsbruck Medical University, Innsbruck, Austria; Groupe Hospitalier Pitié-Salpêtrière, Paris, France; University Hospital of Leuven, Leuven, Belgium; Deutsches Herzzentrum Berlin, Berlin, Germany). The study was appropriately registered (clinicaltrials.gov identifier NCT00761787) and was conducted in accordance with the declaration of Helsinki and Good Clinical Practices [4]. Patients were enrolled at any time post-transplantation after giving a written informed consent to the performance of EMB as part of routine care, the collection of blood samples for research purposes and the use of clinical data [10]. All authors had access to the study data and reviewed and approved the final manuscript.

### Study design and patients

The CARGO II, a prospective observational multi-center trial, was carried out at 17 academic centers (13 from Europe and 4 from North America). Between May 2005 and February 2009, the study enrolled 737 new and existing heart transplant patients who were ≥18 years old and received post-transplant care at the participating centers. Patients who were enrolled in a double blind trial investigating immunosuppressive drugs were excluded from the study. During the study, data on clinical status, EMB grades, echocardiography results and blood for GEP testing were collected at routine surveillance visits. After the last study visit, all participating centers were asked to collect further outcome data on all previously enrolled study subjects. This follow-up case report form included information on the patient’s vital status and causes of death (e.g. infection, malignancy, renal failure or cardiac related), re-transplantation and events of graft failure (see outcome measures).

For this report, we utilized a case cohort study design, a powerful choice for follow-up studies of multiple event types of interest, particularly in the field of human genomics [11]. We selected samples from 12 centers that provided clinical status and events data up to three years following the last study visit. 

### Surveillance testing

The heart transplant recipients enrolled in the study underwent long-term rejection surveillance, with or without EMB. With regards to GEP testing, prepared, frozen blood samples were shipped in standardized packages to the Clinical Laboratory Improvement Amendments (CLIA) certified laboratory (CareDx, Brisbane, California) where the GEP testing was performed (Additional file 1). The AlloMap test has been described in detail previously [7]. Briefly, the test evaluates the expression level of 11 informative genes and 9 genes used for control and normalization [8]. The results are reported as a single GEP score. The test score is associated with a NPV that estimates the probability that a patient is *not* experiencing ≥2R acute cellular rejection [2]. For example, a GEP test score of 32 is associated with a NPV of 98.0 % at 2–6 months post transplant and 99.0 % at more than 6 months post-transplant.

### Outcome measures and study groups

The composite primary clinical outcome of this study was an event of death from any cause, re-transplantation or graft failure. Graft failure was defined as a clinically significant hemodynamic compromise (left ventricular ejection fraction ≤40 %, use of hemodynamic support such as inotropic medications and/or mechanical assist device, hospitalization for treatment of graft failure or dysfunction).

**Fig. 1:**
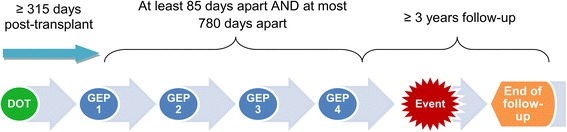
Timing of four serial gene expression profiling scores to predict future clinical events. Four gene expression profiling (GEP) scores were collected beginning day 315 post-transplant. The first and the fourth GEP score were at least 85 days apart and at most 780 days apart. Clinical events were observed ≥ 3 years after the last available score (GEP 4). Therefore, the overall follow-up was up to 6 years (if the patient enrolled in the beginning of 2006 and had a clinical follow-up in late 2011). DOT: date of transplant

The event cohort consisted of all patients who had an event ≥315 days post-transplant. Previous studies have reported that a mean of 3.5 [10] or 4.4 [12] tests/patient have been performed within a 2 years span. Therefore, for the composite event group we included patients who had at least four GEP serial scores prior to the first event. For the control group, we required patients to have at least four serial GEP scores and remain free of an event, before the end of the observation span of study. We also included only the GEP scores that were collected ≥315 days post-transplantation, because of the known upward drift of the mean GEP scores in the first 2 to ~12 months post-transplantation [12]. In an analysis of the population of heart transplant recipients receiving commercial GEP surveillance testing (32,043 GEP tests from 9272 patients), the mean GEP scores increased from an average of 21 at two months to 29 at ~12 months post-transplant. In the next few years beyond ~12 months, the population’s mean GEP score remained stable [12]. Furthermore, we included all clinical events from all patients who had the required 4 serial GEP scores. The time interval between the first and the fourth GEP test visits was at least 85 days apart and at most 780 days apart (Fig. 1).

**Fig. 2:**
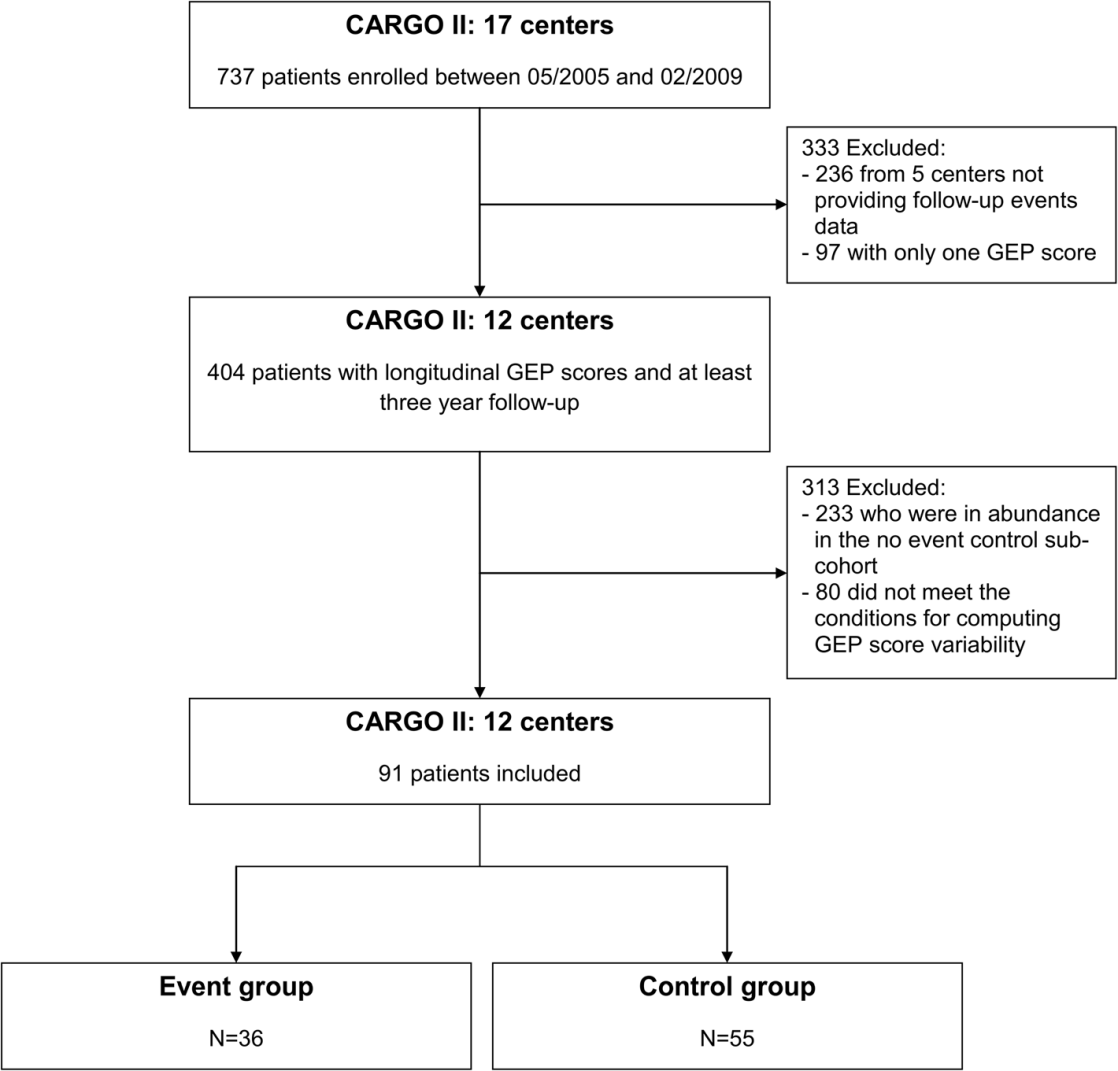
Creation of study cohort using the Cardiac Allograft Rejection Gene Expression Observational (CARGO) II study database. Of 737 patients enrolled in CARGO II study, 171 had at least two gene-expression profiling (GEP) scores and three year follow-up data available. For our cohort, we finally selected 91 patients with at least four serial gene expression profiling scores. These patients were assigned to either an event group or a control group

Of the 737 patients enrolled in the CARGO II study, 333 were excluded (236 from 5 centers not providing follow-up events data and 97 with only one GEP score). Of the 404 remaining patients with longitudinal GEP scores and at least a 3 year follow-up, 288 patients did not have events and were suitable to be controls, but as these were in abundance, we randomly chose 55 patients to be controls and excluded 233. The final cohort of 91 patients thus included all 36 patients who experienced at least one of the predefined clinical events and were included in the event group and 55 patients without events serving as the control group (Fig. 2). The control patients were balanced to the event group with respect to the number of study visits.

GEP score variability (AlloMap score variability, AMV) was defined as the standard deviation of the four consecutive scores collected ≥315 days post-transplantation. For this calculation, the individual scores used were the direct output of the GEP linear discriminate algorithm (LDA) prior to the step that transforms the LDA score to the non-linear GEP score that fits within the 0–40 scale used for the AlloMap report (Additional file 2). The detailed computations of the GEP score variability are presented in Additional file 3.

### Statistical methods

All statistical analyses were performed using R software^©^, 2015, version 3.2.0 (The R Foundation for Statistical Computing, Vienna, Austria), according to the study-group assignment. The area under the receiver operating characteristics curve (AUC ROC) was used to evaluate the prognostic utility of GEP score variability in discriminating between low- and high-risk patients with respect to the occurrence of future events. Sensitivity and specificity were determined for all GEP score variability values between 0.1 and 2.1. Using observed event rates from the CARGO II study, estimates and confidence intervals for PPV and NPV were computed. Confidence intervals (CI) for sensitivity and specificity were calculated using the Clopper-Pearson exact method. Confidence intervals for AUC ROC, NPV and PPV were calculated using re-sampling methods. Demographic variables were compared using the two-sample t-test (age) and two-tailed Fisher’s exact test (categorical variables). The comparisons of days between visits were done using the Wilcoxon test and were reported in terms of medians and inter-quartile range (IQR).

## Results

### Patients

The baseline characteristics of patients were well matched between the two study groups (Table 1). The median number of days from the fourth test to an event was 391 (IQR 376) days.

**Table 1:** Baseline characteristics of study patients

Characteristics	Event group (*n* = 36)	Control group (*n* = 55)	*P* values
Age , years, mean (standard deviation)^a^	53.5 (13.8)	54.4 (13.1)	0.759
Male sex, n (%)	29 (81)	46 (84)	0.781
Race, n (%)			
Caucasian	34 (94)	54 (98)	0.560
Other	2 (6)	1 (2)	0.560
Indication for cardiac transplantation, n (%)			
Coronary Artery Disease	15 (42)	18 (33)	0.504
Idiopathic Cardiomyopathy	14 (39)	30 (55)	0.198
Valvular Heart Disease	2 (6)	3 (5)	1.000
Acute Myocarditis	1 (3)	2 (4)	1.000
Congenital Heart Disease	1 (3)	0 (0)	0.396
Other indications	3 (8)	3 (4)	0.381
Use of ventricular assist device before transplantation, n (%)	6 (17)	13 (24)	0.599
Induction therapy (any), n (%)	28 (79)	45 (82)	0.784
Cytomegalovirus serology (IgG) status, n (%)			
Donor and Recipient Positive	13 (36)	13 (24)	0.239
Donor and Recipient Negative	7 (19)	9 (16)	0.781
Donor Positive and Recipient Negative	3 (8)	6 (11)	1.000
Donor Negative and Recipient Positive	6 (17)	14 (25)	0.439
Unknown	7 (19)	13 (24)	0.797

*P* values were calculated using two-tailed Fisher’s exact test unless indicated otherwise

^a^Two-sample t-test

In the event group, the median interval between transplant and the first GEP test eligible for the GEP variability computation was 1298 (IQR 1975) days; in the control group the median interval was 794 (IQR 1147) days, *P* = 0.351. In the event group and the control group, the last test was performed at a median of 1489 (IQR 2005) and 1119 (IQR 1262) days post-transplant respectively, *P* = 0.399. There was no statistical difference between the groups in number of days between the first and the last GEP test; in the event group, the median was 336 days (IQR 147) and in the control group, the median was 315 (IQR 108) days, *P* = 0.858.

### GEP score variability

**Fig. 3:**
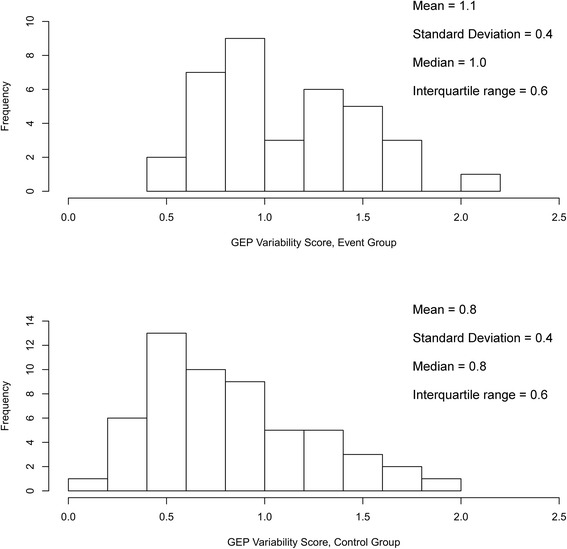
Distribution of gene expression profiling scores of all study patients assigned to the event group and the control group

Of all patients, 63 % (57/91) had a GEP score variability ≤1.0; 16 % (15/91) of patients had a score variability ≤0.5. The majority of patients had GEP score variability ranging from 0.5 to 1.5 (78 %, 71/91). The distribution of the GEP scores in both study groups is shown in Fig. 3.

**Fig. 4:**
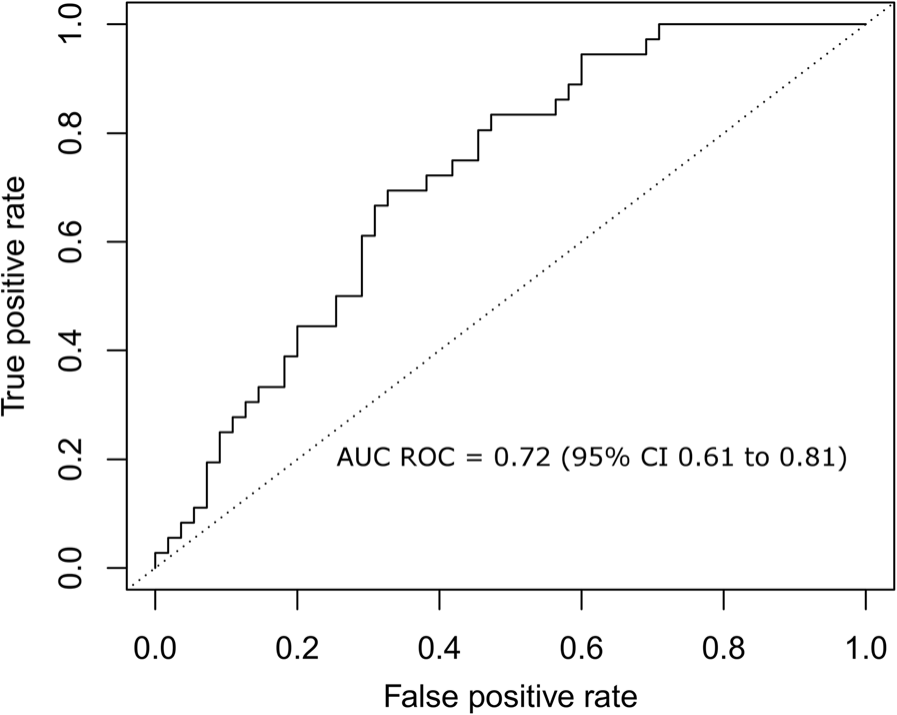
The area under the receiving operator characteristics (AUC ROC) for AlloMap score variability to predict future clinical events (death from any cause, re-transplantation or graft failure)

The estimated prevalence of events in our data set was 17 %. The AUC ROC for the GEP score variability to predict a composite, clinically significant event was 0.72, 95 % CI from 0.61 to 0.81 (Fig. 4).

The GEP score variability NPV and PPV performance characteristics are shown in Table 2. In our study, the NPV increased from 87.4 % (95 % CI 80.1-92.9) at a GEP score variability of 1.0 to 97 % (95 % CI 91.4-100.0) at a GEP score variability of 0.6. The PPVs for the same score decreased from 26 % (95 % CI 14.8-41.8) at a GEP score variability of 1.0 to 23.3 % (95 % CI 15.7-33.0) for a GEP score variability of 0.6. For a GEP score variability cutoff of 1.5, the estimated PPV is 35.4 % (95 % CI 13.5-75.8).

**Table 2:** GEP variability performance results

Variability Score	Scores ≤ Threshold %	PPV %	NPV %	Sensitivity %	Specificity %
		(95 % CI)	(95 % CI)	(95 % CI)	(95 % CI)
0.1	0.0	17.0 (−−)	-	100.0 (90.3–100)	0.0 (0.0–6.5)
0.2	1.1	17.2 (11.6–23.8)	100.0 (−−)	100.0 (90.3–100)	1.8 (0.0–9.7)
0.3	3.3	17.8 (12.3–24.7)	100.0 (−−)	100.0 (90.3–100)	5.5 (1.1–15.1)
0.4	7.7	19.0 (13.1–26.9)	100.0 (−−)	100.0 (90.3–100)	12.7 (5.3–24.5)
0.5	16.5	22.0 (15.2–30.8)	100.0 (−−)	100.0 (90.3–100)	27.3 (16.1–41.0)
0.6	24.2	23.3 (15.7–33.0)	97.0 (91.4–100.0)	94.4 (81.3–99.3)	36.4 (23.8–50.4)
0.7	33.0	23.2 (15.4–33.7)	92.8 (85.4–97.8)	83.3 (67.2–93.6)	43.6 (30.3–57.7)
0.8	42.9	25.2 (16.3–37.1)	91.4 (84.2–96.5)	75.0 (57.8–87.9)	54.5 (40.6–68.0)
0.9	56.0	29.7 (18.5–46.0)	90.3 (83.9–95.2)	63.9 (46.2–79.2)	69.1 (55.2–80.9)
1.0	62.6	26.0 (14.8–41.8)	87.4 (80.1–92.9)	50.0 (32.9–67.1)	70.9 (57.1–82.4)
1.1	68.1	27.8 (15.3–46.5)	87.0 (80.4–92.4)	44.4 (27.9–61.9)	76.4 (63.0–86-8)
1.2	71.4	29.9 (16.3–51.0)	87.0 (80.3–92.2)	41.7 (25.5–59.2)	80.0 (67.0–89.6)
1.3	76.9	29.4 (14.6–53.4)	86.0 (79.3–91.3)	33.3 (18.6–51.0)	83.6 (71.2–92.2)
1.4	83.5	31.9 (13.5–63.2)	85.3 (78.7–90.6)	25.0 (12.1–42.2)	89.1 (77.8–95.9)
1.5	87.9	35.4 (13.5–75.8)	84.9 (78.5–90.1)	19.4 (8.2–36)	92.7 (82.4–98.0)
1.6	92.3	29.4 (5.9–100.0)	83.9 (77.1–89.4)	11.1 (3.1–26.1)	94.5 (84.9–98.9)
1.7	94.5	31.9 (0.0–100.0)	83.7 (77.1–89.1)	8.3 (1.8–22.5)	96.4 (87.5–99.6)
1.8	97.8	23.8 (−−)	83.2 (76.6–88.6)	2.8 (0.1–14.5)	98.2 (90.3–100.0)
1.9	98.9	100.0 (−−)	83.4 (77.0–88.7)	2.8 (0.1–14.5)	100.0 (93.5–100.0)
2.0	98.9	100.0 (−−)	83.4 (77.0–88.7)	2.8 (0.1–14.5)	100.0 (93.5–100.0)
2.1	100.0	--- (−−)	83.0 (76.6–88.4)	0.0 (0.0–9.7)	100.0 (93.5–100.0)

*CI* confidence interval, *GEP* gene expression profiling, *NPV* negative predictive value, *PPV* positive predictive value

In the event group 58 % (21/36) of patients died, 31 % (11/36) experienced graft failure and 11 % (4/36) underwent cardiac re-transplantation.

## Discussion

The most important finding of this study is that the GEP score variability may be useful in estimating the probability of future events of death, re-transplantation or graft failure in heart transplant recipients undergoing surveillance with GEP testing ≥315 days post-transplantation. A low variability of sequential GEP scores (≤0.6), which was found in 24.2 % of the study population, rendered NPVs of ≥97.0 % (Table 2), indicating clinical utility of GEP score variability in the identification of patients at a low risk for future clinical events of greatest concern. This finding may have important clinical implications in the longer-term management of heart transplant recipients as the low risk patients may be good candidates for optimization (i.e. reduction) of their immunosuppressive drugs. Consequently, we speculate that this may reduce the rate of unwanted adverse effects associated with chronic immunosuppression, particularly infections and nephrotoxicity. We believe that future GEP studies should focus on personalization of long-term immunosuppressive therapy and potential improvements in long-term outcomes of heart transplant recipients.

Using peripheral-blood specimens, the AlloMap gene expression test translates the complex gene expression patterns of the mononuclear blood cells into a single score (0–40) using a proprietary algorithm (Additional file 2). The risk of rejection is considered to be higher immediately after transplantation and lower by six to 12 months post-transplant [13]. In the IMAGE study, the use of a single gene expression profiling score (below the 34 threshold) to assess a risk of acute cellular rejection in clinically stable patients six months post cardiac transplant resulted in a significant decrease in the number of EMB performed. This reduction was achieved without adversely affecting patient outcomes [7]. More recently, another randomized clinical trial has shown similar results in patients who began the AlloMap surveillance two months post-transplant, which is considered a higher risk rejection time frame [14]. Primarily based on the IMAGE study results, the International Society of Heart and Lung Transplantation (ISHLT) suggested that a single AlloMap score can be used to rule out the presence of acute cellular rejection of ≥ 2R in selected low-risk patients [2]. In contrast, the GEP score variability analysis was performed to aid clinicians in assessing the future risk of a significant clinical event (death, re-transplantation or graft failure). In the field of transplant cardiology, there is a paucity of biomarkers that can reliably predict which patients are at risk of adverse clinical events [15]. There is a growing need to develop evidence-based personalized strategies to optimize immunosuppressive therapy, improve long-term outcomes and reduce complications [15]. Therefore, and in addition to the individual GEP score and clinical assessment, the GEP score variability may provide useful complementary information that may help personalize long-term care of heart transplant recipients.

Deng and colleagues computed GEP score variability and performed post-hoc analyses on IMAGE patients who had at least two single GEP scores before an event or study end [10]. In that study, multivariate analyses revealed that only GEP score variability was significantly associated with future clinical events; gender, race, age and cytomegalovirus serological status were not. In the cohorts presented in this article, we prospectively analyzed clinical outcome data of the CARGO II patients who had four GEP scores preceding a first clinical event (event group) or had 4 sequential GEP scores without a subsequent event (control group). In addition to the fact that a mean of 3.5 or 4.4 tests are performed within a two year surveillance interval [10, 12], our decision to use four serial GEP scores was based on calculating 85 % probability of being within twofold of true variance (using 3 scores yields 76 % probability). Although we included two more GEP scores than Deng and colleagues in our calculation, the formula for computing GEP score variability remained the same (Additional file 3) [10]. Importantly, enrollment in the current study began in 2005. The latest follow-up clinical data was provided by the end of 2011, allowing us to predict a significant clinical event up to 6 years following heart transplantation. Finally, we also computed NPV, PPV, sensitivity and specificity for the range of GEP score variability between 0.1 and 2.1.

Our results must be interpreted while considering the limitations of this study. First, our composite primary clinical outcome is a mixture of clinical conditions including subtypes of events that were rare (e.g. only four events of re-transplantation). In the future, we would aim to collect larger numbers and/or more specific subtypes of endpoints with sufficient power to be separately analyzed (e.g. deaths from infections or cancers due to long term sequelae of over-immunosuppression). Additionally, the expression levels of the informative genes used to compute GEP scores in some cases may reflect a feature of the status of the recipient’s immune system that is not directly associated with the allograft [10]. Previous studies have indicated that prednisone doses above 20 mg/day may reduce the GEP score by inhibiting the expression of IL1R2, FLT3, and ITGAM genes [10]. Most recently, it has been reported that acute cytomegalovirus infections may be associated with increased GEP scores in the absence of acute cellular rejection [16]. Our retrospective analysis of this case-cohort study may introduce potential, unintentional bias in the selection of patients. To minimize selection bias, we included all patients who met our predetermined definition of clinical events in the event group. All other non-event patients from the CARGO II study who had four GEP scores were assigned to the control group. Moreover, one may argue that the limited sample size (36 in the event group and 55 in the control group) and the imbalance between the number of patients in each study group could affect our findings. The use of unequal assignments ≤ 3:1 between study groups does not significantly reduce the power of the study [17]. Also, the baseline characteristics of study patients were well-matched at entry.

In our study, the performance of the GEP score variability in predicting outcomes for patients who were monitored with GEP testing ≥315 days post-transplantation (AUC ROC of 0.72, 95 % CI 0.61 to 0.81) is similar to the performance of the established single GEP score in ruling out acute cellular rejection for patient samples ≥2-6 month post-transplant (AUC ROC 0.71, 95 % CI 0.56 to 0.84). The limited PPV of the GEP score variability (Table 2) will undoubtedly be compared with interpretation of EMB findings for acute cellular rejection of cardiac transplants. In another report, observed agreement between local and central pathologists for EMB scoring was 28 % for grade ≥ 2R (2004 ISHLT grading system) [4]. Despite the ISHLT simplification of the 1990 grading classification, suboptimal EMB readings are still common [4]. Furthermore, the GEP score variability may predict future clinical events rather than be used for ruling out acute cellular rejection at the time of a test. In this study, the estimated PPVs do not exceed 36 % for variability values of up to 1.7. This limitation of the PPV is in part attributable not only to the underlying sensitivity of this measure, but to the low prevalence of events in the study population. Since the NPV exceeds 97 % for variability values of ≤0.6, this test result may be best suited for use to “rule-out” rather than for use to “rule in” the likelihood of a future clinical event (Table 2). Specific NPV and PPV values (Table 2) are provided to aid clinicians in estimating the likelihood of death, re-transplantation and graft dysfunction occurring in patients beyond 315 days post-transplant. However, the clinician may choose how to use the particular GEP score variability threshold based on a practical experience and make an associated clinical interpretation based on overall clinical presentation of the individual patient. Based on the results of this study, we suggest use of GEP score variability to predict a future composite clinical event (death from any cause, re-transplantation or graft failure) only for GEP tests collected at ≥315 days post-transplant. For example, the patients with GEP score variability ≥1.6 (approximately 8 % of the CARGO II population) may be at a higher risk of experiencing an event (PPV ~30 %; Table 2). These patients may be candidates for more vigilant surveillance and appropriately timely interventions. On the other hand, patients with a low GEP score variability, particularly scores ≤ 0.6 (approximately 25 % of our population) may be at a low risk of experiencing a clinical event (NPV ≥97 %) and may be potential candidates, depending on the full clinical circumstances in each case, for reduced immunosuppression.

## Conclusions

The GEP score variability may be used in estimating the likelihood of events of death, re-transplantation or graft dysfunction occurring in patients beyond 315 days post-transplant and thus may aid physicians in identifying heart transplant recipients who may need more or less intensive further surveillance. Prospective studies of GEP score variability including a larger number of patients and events from the Outcome AlloMap Registry are desirable in the future.

## Additional files

Additional file 1: Manufacturing of Gene expression profiling assay, phlebotomy and standardized processing of frozen blood samples in the Clinical Laboratory Improvement Amendments (CLIA) certified laboratory (CareDx, Brisbane, California) where the GEP testing is performed. (MP4 146998 kb) Additional file 2:
**Algorithm to compute the gene-expression profiling (GEP) score.** (DOCX 18 kb) Additional file 3:
**Gene expression profiling (GEP) score variability computation.** (DOCX 17 kb)

## Abbreviations

AMV: AlloMap variability
AUC ROC: Area under the receiver operator characteristics curve
CARGO: Cardiac allograft rejection gene expression observational
CI: Confidence interval
CLIA: Clinical Laboratory Improvement Amendments
EMB: Endomyocardial biopsy
GEP: Gene expression profiling
IMAGE: Invasive monitoring attenuation by gene expression profiling
ISHLT: International Society of Heart and Lung Transplantation
NPV: Negative predictive values
PPV: Positive predictive values
