# Supplementary material for: Performance of gene-expression profiling test score variability to predict future clinical events in heart transplant recipients
Source: BMC Cardiovasc Disord. 2015 Oct 9;15:120. doi: 10.1186/s12872-015-0106-1 (PMC4600291; doi:10.1186/s12872-015-0106-1)
Supplement: Additional file 2: — Algorithm to compute the gene-expression profiling (GEP) score. (DOCX 18 kb) [file 12872_2015_106_MOESM2_ESM.docx]

**Addition File 2 Algorithm to compute the gene-expression profiling (GEP) score**

The AlloMap gene expression test translates the complex gene expression patterns of the mononuclear blood cells into a single score (0-40) using a proprietary algorithm. The final informative classifiers used to compute the GEP (AlloMap) score (3 metagenes and 4 individual genes) are listed in the brackets below. First, using the GEP linear discriminate algorithm (LDA), the individual LDA score (x) is calculated using the formula:

x = 105.96

+ 1.413 * C_T_{IL1R2,FLT3,ITGAM}

– 1.639 * C_T_{MIR,WDR40A}

+ 0.344 * C_T_{PF4,G6b}

– 1.340 * C_T_{ITGA4}

– 0.838 * C_T_{PDCD1}

– 0.684 * C_T_{RHOU}

– 0.739 * C_T_{SEMA7A}

C_T_ = threshold cycle

Then, the individual LDA score is converted to a single GEP score (0-40) using the following formula:

$$Gene Expresion Profiling (GEP) Score=40\times\frac{e^{(0.234+0.408\times LDA)}}{1+e^{(0.234+0.408\times LDA)}}$$

*x* = LDA score

Finally, the GEP score is truncated and reported as an integer.

Higher values of *x* and *GEP* indicate a higher risk of current acute cellular rejection.

# Reference: Deng MC, Eisen HJ, Mehra MR, Billingham M, Marboe CC, Berry G et al.. Noninvasive discrimination of rejection in cardiac allograft recipients using gene expression profiling. *Am J Transplant.* 2006; 6:150-60.
