# Supplementary material for: Performance of gene-expression profiling test score variability to predict future clinical events in heart transplant recipients
Source: BMC Cardiovasc Disord. 2015 Oct 9;15:120. doi: 10.1186/s12872-015-0106-1 (PMC4600291; doi:10.1186/s12872-015-0106-1)
Supplement: Additional file 3: — Gene expression profiling (GEP) score variability computation. (DOCX 17 kb) [file 12872_2015_106_MOESM3_ESM.docx]

**Additional File 3 Gene expression profiling (GEP) score variability computation**

The following steps are taken to calculate the GEP score variability:

1. Collect the 4 eligible individual scores within the parameters described in this paper. These 4 scores are the direct output of the GEP linear discriminate algorithm (LDA) prior to the step that transforms the individual LDA score to the non-linear GEP score (range 0-40) used for the AlloMap report.
2. Calculate GEP score variability defined as the standard deviation of the four LDA scores using the following formula:

$$\sigma=\sqrt{\frac{1}{N-1}\sum_{i=1}^{N} {(x_{i}-\mu)}^{2}}$$

*x_i_ =* 4 LDA scores

μ = mean of 4 LDA scores

# Reference: Deng MC, Elashoff B, Pham MX, Teuteberg JJ, Kfoury AG, Starling RC, et al.. Utility of gene expression profiling score variability to predict clinical events in heart transplant recipients. *Transplantation.* 2014; 97:708-14.
